# Hospitals

**Published:** 1877-03

**Authors:** 


### Hospitals.

### ST. LUKE'S HOSPITAL.

### FIVE CASES OF INTERNAL HÆMORRHOIDS TREATED BY THE CLAMP AND ACTUAL CAUTERY.

(Under the care of JNO. E. OWENS, M.D.)

Case I. Internal piles, complicated with hypertrophy of the uterine cervix and painful ulceration at the anus.

Mrs. W., aged 44 years, was admitted to St. Luke's Hospital Jan. 10, 1874. Ten days after admission Prof. DeLaskie Miller amputated the neck of the uterus for hypertrophy. The patient had suffered from internal piles for seventeen years. Her health was seriously impaired from loss of blood from the rectum, from pain, and other distress occasioned by a cluster of purple hæmorrhoidal tumors, which protruded on all occasions. Feb. 1, I applied the clamp and cautery seven times to as many tumors. A tag of skin, inflamed, œdematous and ulcerated was at the same time removed with the scissors. This patient did not recover with the promptitude usually noticed after operations for piles, and the case, in this respect, verifies Allingham's statement, that patients with uterine complications recover slowly from operations upon the rectum. She experienced a sense of obstruction while at stool; pain, more or less severe, began with defecation, and continued from one to six or eight hours after the completion of the act. The cut surface at the site of the tag of skin above referred to had become (Mch. 6) a painful ulcer, the upper margin of which barely touched the verge of the anus. As the ulceration did not pass within the anus,I hoped to be able to effect a cure without incision. Suffice to say that I failed with ointments, lotions, separation of its surfaces, nitrate of silver, rest, etc. Finally, April 22, I incised the ulcer and that part of the sphincter muscle contiguous to it. This relieved the characteristic pain, the ulcer healed, and the patient was discharged May 19th. Eleven weeks after the operations for hæmorrhoids an opportunity presented itself for the examination of the rectum with the object of noting its condition after the application of the actual cautery. The lower part of the rectum was less distensible than the normal rectum; its mucous membrane was smooth; firm, irregular scars were felt in and apparently beneath the mucous membrane, but they were not visible, and could only be located by the sense of touch. The patient has continued well to this date, Feb. 8, 1877.

Case II. T. O'B., aged 50 years, had suffered, in consequence of internal piles, for fourteen years. The tumors bled more or less every week, and protruded daily. He had some pain, frequently in the anus, and also over the sacrum, and about the hips. Thus afflicted, he was obliged to work, to ward off starvation. Having been admitted to the hospital, the operation with clamp and cautery (Leplié cautery clamp*) was

* See Braithwaite's Retrospect, July, 1874, page 118.

performed Jan. 2, 1875. Six applications were made. The patient was put to bed, and no dressings were used till Jan. 8th, when the swollen mucous membrane of the rectum, as is usual, protruded somewhat. This yielded to dressings of cold water, together with moderate pressure. The pain following the operation was of light degree. The bowels were kept constipated with laudanum for three or four days. The patient was discharged cured in eleven days from the date of the operation.

CASE III. H. D. G., aged 40 years, was admitted March 4, 1875. In this case the clamp and cautery were applied seven times, March 9, 1875; the patient was then put to bed and cold water dressings employed. Anodines were not indicated. March 10, A. M., an injection of two ounces of warm castor oil was ordered, and at 4 o'clock P. M. two drachms. The bowels moved at 7 o'clock P. M., and also on the morning of the 11th. The patient did not suffer. I have no note of the duration of the man's affliction, but the case was one of unusual severity. In consequence of hæmorrhage and pain he was absolutely unable to follow his trade, that of harness making. He had tried many so-called remedies without benefit. March 12, the third day after the operation, the patient expressed himself as feeling better than he had done in a long time, and started for his home in Indiana. I afterwards received a letter from him confirming the satisfactory result of the operation.

CASE IV. L. A. S., aged 44 years, a shoemaker, was admitted March 21, 1876. He had had internal hæmorrhoids for twenty years. During the last seven years the tumors had not bled, but previous to that time the hæmorrhages were of frequent recurrence. March 22, two applications of the clamp and cautery were made. The patient was put to bed without dressings. March 25, the bowels responded to injections of warm castor oil. The protrusions of swollen rectal mucous membrane was very slight, not greater in size than a pea. March 29, the seventh day after the operation, the patient went to his home in Illinois, about five hours ride from Chicago.

CASE V. M. M., aged 45 years, came to the hospital Aug. 11, 1876. The same day three large hæmorrhoids were disposed of by means of the clamp and cautery. The subsequent swellings and protrusions of the mucous membrane were slight. The patient left the hospital Aug. 15, the fourth day after the operation.

---

### COOK COUNTY HOSPITAL.

### COMPOUND FRACTURE OF THE LEG; AMPUTATION; HOSPITAL GANGRENE; AMPUTATION OF THE THIGH; LIGATION OF THE EXTERNAL ILIAC ARTERY; RECOVERY.

R. V., a German carpenter, aged 44 years, admitted to Cook County Hospital May 6, 1872, states that, attempting to get on a street car while in motion, he fell under the wheels. On examination, it was found that the right leg was crushed, most of the lower third being involved. There were three small openings of the skin, but not much hæmorrhage. The main vessels were intact. The patient being opposed to having the leg amputated, it was decided to place it in a fracture box and to make light extension. Carbolic acid lotions were applied, and morphia was given to relieve pain.

May 21. Extensive gangrene of the soft tissues ensued, but finally a line of demarcation formed in about the middle of the leg. The patient was then willing to submit to whatever the surgeons decided upon. Dr. Freer amputated the leg at the junction of the middle and upper thirds. Circular operation.

Considerable inflammation and induration remaining below the knee, it was decided not to close the flaps with sutures, but simply to draw them together with adhesive straps.

July 27. Phlegmonous inflammation and protracted suppuration had followed the operation. The loss of the tissues and the consequent retraction of the integument had been so great as to leave no covering to the bone. Therefore the stump has to be reamputated.

July 31. Erysipelas extending up the thigh. The tincture of iodine, and cloths saturated in a solution of the acetate of lead were applied.

Aug. 20. Discharged from the hospital at his own request.

Oct. 1. Readmitted to hospital, because the stump failed to heal. The acid nitrate of mercury was applied.

Oct. 18. The stump acquired a healthy granulating surface. Three points were inserted by grafting.

Oct. 28. The patient has not been feeling well for several days, having considerable fever, thirst, loss of appetite, coated tongue, and great pain in the stump. The latter has assumed a gangrenous aspect in the granulated portion. The edges are being eaten away very fast. Quinine is prescribed in four-grain doses three times daily, and carbolic acid is applied to the stump.

Nov. 7. The sloughing has ceased, and the patient is doing moderately well.

Nov. 10. Stump clean, although it gives him pain. Appetite poor. Soreness of the throat complained of. Upon examination a small, deep, well-defined ulcer was discovered on the right side of the soft palate. Tincture of iron and chlorate of potash were ordered as a wash.

Jan. 28, 1873. Reamputation of the stump by Dr. Powell, three-fourths of an inch of bone being removed. There was considerable hæmorrhage. The wound was left open for an hour or so. When nearly all bleeding had stopped it was stitched together, but had to be reopened in a short time and packed with sponges soaked in persulphate of iron solution.

Jan. 29. The sponges were removed and the wound was packed with lint soaked in carbolized oil.

Feb. 3. A slight hæmorrhage took place this morning. Stump very tender. A violent hæmorrhage about 4 P. M., the blood coming from the popliteal artery, the tissues being very soft and the artery retracted. A tourniquet was applied, and the open end packed with sponge soaked in a solution of the persulphate of iron.

Feb. 4. A little bleeding again this morning. 3 P. M. Dr. Powell amputated the thigh at the junction of the lower and middle thirds. There was but very little hæmorrhage. The cut surfaces were closed at the end of an hour by sutures. About an hour later slight bleeding occurred, enough to stain the dressings.

Feb. 5. About one o'clock this morning a little more hæmorrhage took place. About three o'clock the stump became very painful, and a short time afterward bleeding again was noticed. About four o'clock the dressings and bed were considerably stained.

Feb. 18. Two weeks to-day since the last operation. All the ligatures except one have come away. About noon, while the patient was resting quietly in bed, he felt a slight twinge in the stump, followed in a few moments by bleeding. The bandage becoming saturated, the hæmorrhage ceased.

Feb. 23. About noon a copious arterial hæmorrhage occurred. Dr. Bogue ligated the common femoral artery about half an inch below Poupart's ligament. The artery appeared to be very friable; for, in passing the ligature, the artery was accidentally opened. Another copious hæmorrhage ensued. The artery was tied both above and below the puncture with a silver wire, the ends of the knot being cut off close. The surface wound was stitched together with silver wire.

Feb. 28. Stump looking well. A slight discharge of healthy pus from the wound over the femoral artery.

March 13. Wound over the femoral is discharging considerably; a little discharge from the inner and outer angles of the stump.

March 20. The upper femoral ligature ulcerated through to the surface of the wound, and was removed.

March 22. The lower knot came away to-day.

March 25. Another hæmorrhage last night from the end of the stump which had healed over, except two minute openings, not much larger than a knitting-needle at the outer angles. The blood came through both openings, perhaps two ounces in all. A wet compress was put over both openings and a bandage around the stump.

March 28. A copious hæmorrhage about two o'clock this morning from the wound in the groin. This is the thirty-fourth day since the artery was tied, and the seventh since the second knot came away. The opening at this time was about two inches in length and half an inch in its greatest depth, and was dressed with lint soaked in alum water. The blood jetted up with such force as to throw the dressing from the wound. The patient says the stream was as large as the tip of his index finger. The house surgeon was at his bedside in five minutes after the first escape of blood. The blood was then running from the wound at both angles. The bed was saturated, the patient lying in a perfect pool of blood, partly clotted. Pressure over the pubic bone arrested all bleeding. Ether was administered, and Dr. Bogue proceeded to tie the external iliac artery, Dr. Powell assisting. The peritoneum was wounded a little during the operation. The ligature, a double silk waxed thread, was passed without any trouble. The ends of the ligature were brought out at the inferior angle of the wound. Four deep and five superficial silk sutures were used in closing the wound. Adhesive plaster, compress and roller were applied. Hot milk punch was administered, and morphia by injection, to relieve pain.

April 2. Stitches removed; wound gaps a little and discharges a good quantity of pus.

April 21. The ligatures of the iliac artery came away this morning; wound looking well.

June 6. Patient has recovered at last to be discharged, 122 days after the amputation of the thigh.

